# Supplementary material for: Comparing mouse and human cingulate cortex organization using functional connectivity
Source: Brain Struct Funct. 2024 May 13;229(8):1913–25. doi: 10.1007/s00429-024-02773-9 (PMC11485145; doi:10.1007/s00429-024-02773-9)
Supplement: Supplementary file 1 — Supplementary file1 (PDF 789 KB) [file 429_2024_2773_MOESM1_ESM.pdf]

Supplementary material for

Comparing mouse and human cingulate cortex organization using functional connectivity

Aran T.B. van Hout[1], Sabrina van Heukelum[1], Matthew F.S. Rushworth[2,3], Joanes Grandjean[1,4]*, Rogier B. Mars[1,2]*

* shared last authors

[1]Donders Institute for Brain, Cognition and Behaviour, Radboud University Nijmegen, Nijmegen, The Netherlands
[2]Wellcome Centre for Integrative Neuroimaging, Nuffield Department of Clinical Neurosciences, John Radcliffe Hospital, University of Oxford, Oxford, United Kingdom
[3]Department of Experimental Psychology, University of Oxford, Oxford, United Kingdom
[4]Department for Medical Imaging, Radboud University Medical Center, Nijmegen, The Netherlands

Correspondence:

Rogier B. Mars
Donders Centre for Cognition
Thomas van Acquinostraat 4
6525 GD Nijmegen
The Netherlands
Rogier.mars@donders.ru.nl

Supplementary material A: Mouse and human cingulate seed areas

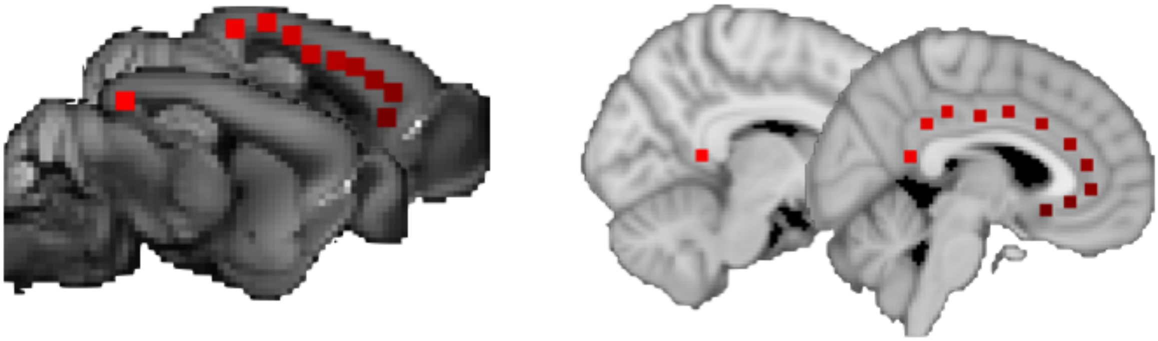

Supplementary material B: Four component ICA solution for mouse tracer data

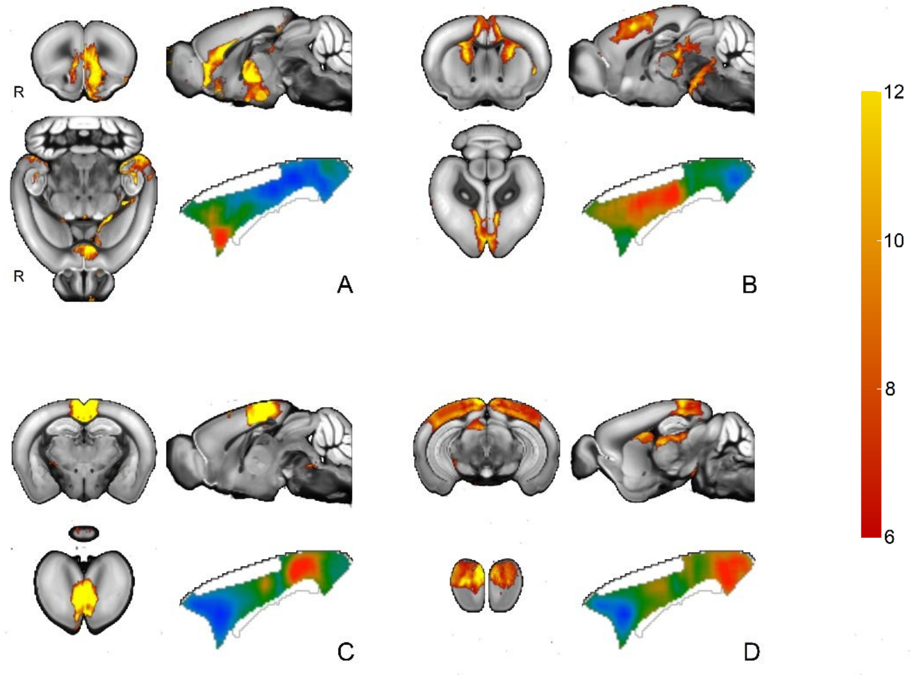

Each mix figure represents the normalised contributions of seeds to the ICA component (blue: low contribution, red: high contribution) The four components include: 25/32 component (panel A), the secondary motor component (panel B), the retrosplenial area component (panel C), and the visual component (panel D).

Supplementary material C: Nine component ICA solution for the mouse tracer data

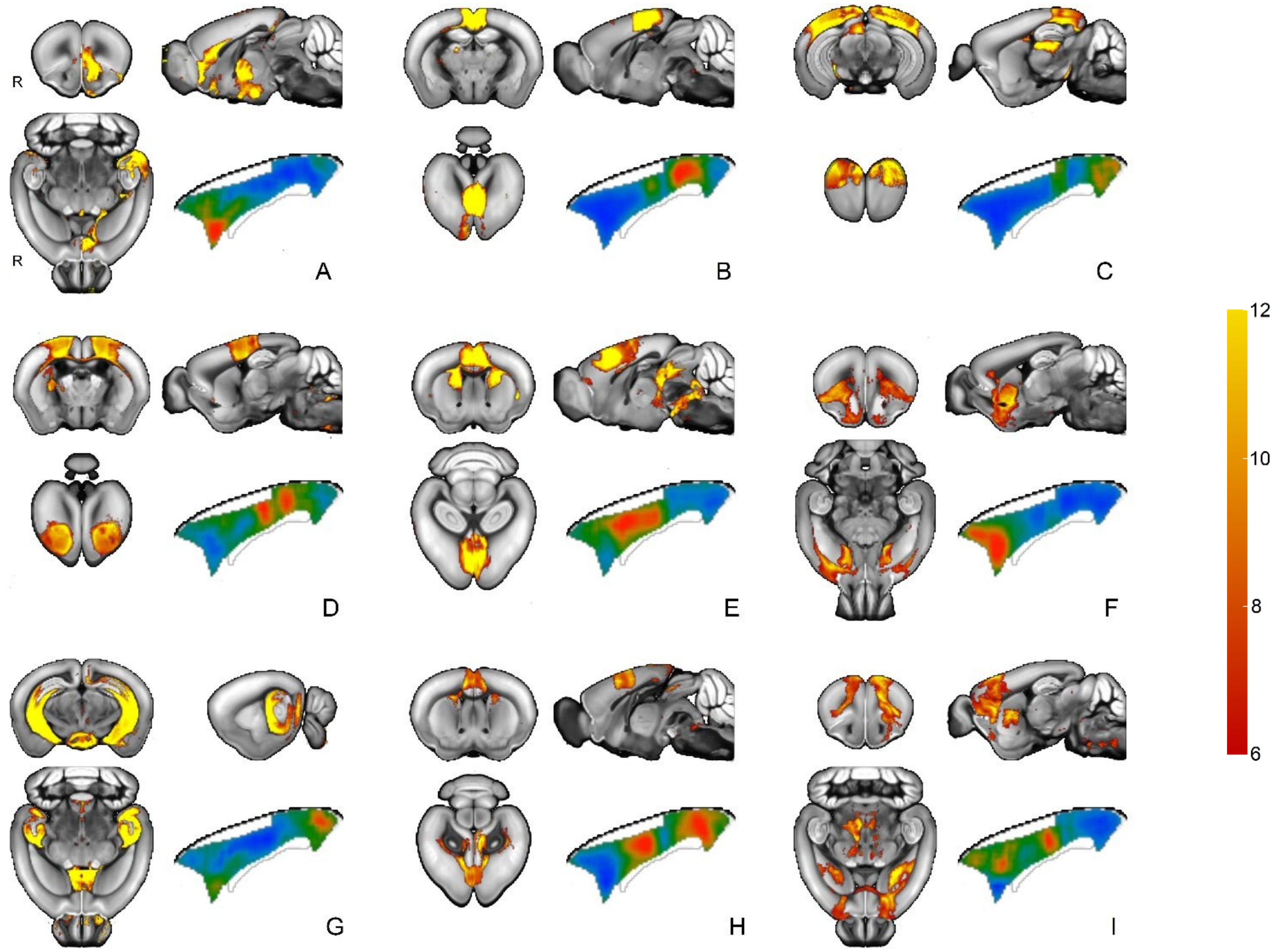

Each mix figure represents the normalised contributions of seeds to the ICA component (blue:low contribution, red: high contribution) The nine components include: area 25/32 component (A), the retrosplenial area component (B), the visual component (C), the secondary motor area component (D), the area 24/24' component (E), orbital/insula component (F), the hippocampal component (G), the area 24' component (H), and the orbital/secondary motor component (I).

Supplementary material D: Additional mouse cingulate seeds resting state fMRI connectivity maps

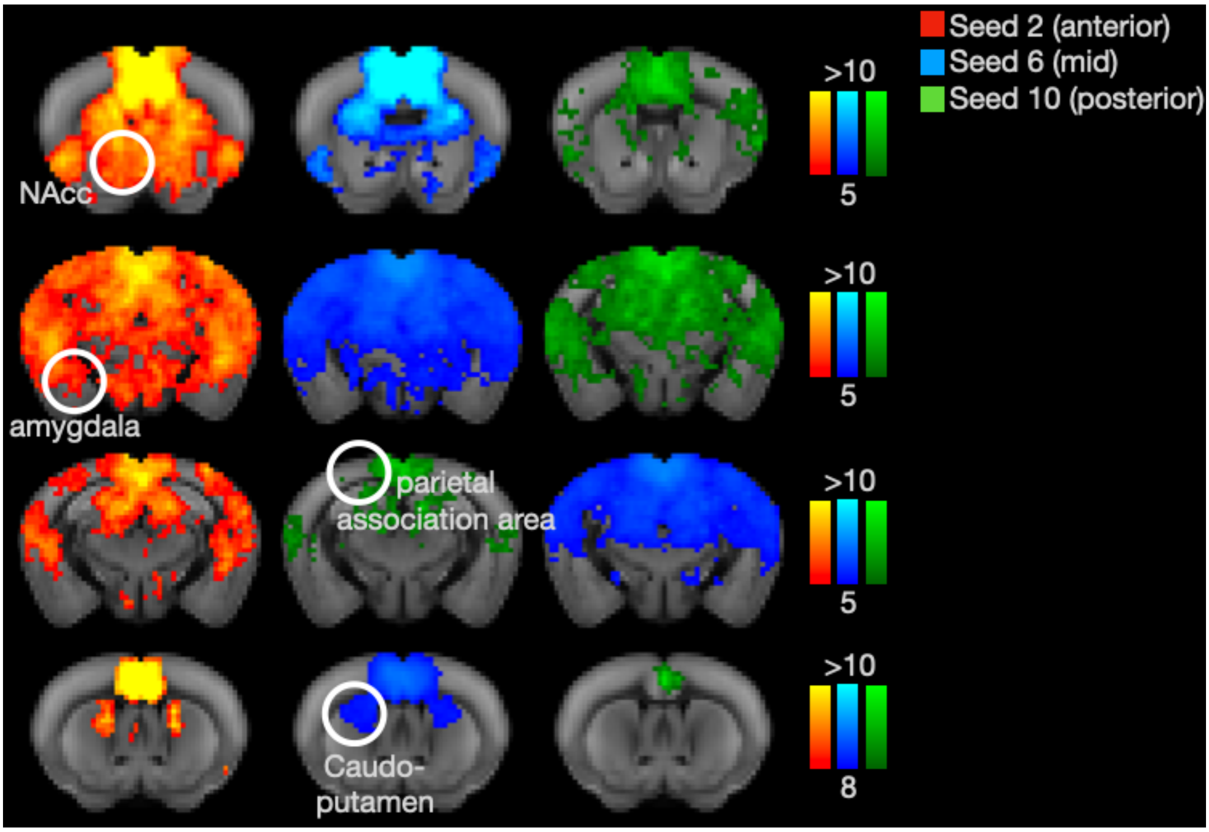

Connectivity of mouse anterior, mid-, and posterior cingulate seeds. Color strengths indicate z-statistics.
